# Identification, Characterization, and Expression Analysis Reveal Diverse Regulated Roles of Three MAPK Genes in *Chlamys farreri* Under Heat Stress

**DOI:** 10.3389/fphys.2021.688626

**Published:** 2021-07-28

**Authors:** Zhi Liu, Xiaoting Huang, Zujing Yang, Cheng Peng, Haitao Yu, Chang Cui, Yuqing Hu, Xuefeng Wang, Qiang Xing, Jingjie Hu, Zhenmin Bao

**Affiliations:** ^1^MOE Key Laboratory of Marine Genetics and Breeding, College of Marine Life Sciences, Ocean University of China, Qingdao, China; ^2^Laboratory for Marine Fisheries Science and Food Production Processes, Pilot Qingdao National Laboratory for Marine Science and Technology, Qingdao, China; ^3^Laboratory of Tropical Marine Germplasm Resources and Breeding Engineering, SANYA Oceanographic Institution of the Ocean University of CHINA, Sanya, China

**Keywords:** mitogen-activated protein kinase, *Chlamys farreri*, heat stress, molecular response, bivalve adaptation

## Abstract

Mitogen-activated protein kinase (MAPK) cascades are fundamental signal transduction modules in all eukaryotic organisms, participating growth and development, as well as stress response. In the present study, three MAPK genes were successfully identified from the genome of *Chlamys farreri*, respectively, named *CfERK1/2*, *CfJNK*, and *Cfp38*, and only one copy of *ERK*, *JNK*, and *p38* were detected. Domain analysis indicated that *CfMAPK*s possessed the typical domains, including S_TKc, Pkinase, and PKc_like domain. Phylogenetic analysis showed that three *CfMAPK*s of MAPK subfamilies exists in the common ancestor of vertebrates and invertebrates. All *CfMAPK*s specifically expressed during larval development and in adult tissues, and the expression level of *CfERK1/2* and *Cfp38* was apparently higher than that of *CfJNK*. Under heat stress, the expression of *CfERK1/2* and *Cfp38* were significantly downregulated and then upregulated in four tissues, while the expression of *CfJNK* increased in all tissues; these different expression patterns suggested a different molecular mechanism of *CfMAPKs* for bivalves to adapt to temperature changes. The diversity of *CfMAPKs* and their specific expression patterns provide valuable information for better understanding of the functions of MAPK cascades in bivalves.

## Introduction

The Zhikong scallop, *Chlamys farreri* (Jones, 1904, also known as Chinese scallop), naturally distributes along the coasts of Northern China, Korea, Japan, and Eastern Russia, which usually live a semisessile lifestyle by attaching itself to rocks and other hard surfaces with byssal threads (Shumway and Parsons, 2016). *C. farreri* is one of commercially important scallops for aquaculture in China (Guo and Luo, 2016). The production of scallops in China showed a declining tendency for the last few years, which was 2.01 million tons in 2017, 1.92 million tons in 2018, and 1.83 million tons in 2019 (China Fishery Statistical Yearbook 2019 and 2020). In recent years, extreme weather especially high temperatures happened frequently worldwide, which threatened the survival of almost all marine organisms. Stress caused by sudden changes in temperature or chronic heat stimuli above optimum conditions can interrupt cellular homeostasis and result in serious deficiency in development and growth and even large-scale death in bivalves, including scallop, mussel, and oyster (Liu et al., 2004; Xiao et al., 2005; Hornstein et al., 2018; Rahman et al., 2019; Zhang et al., 2020).

High temperature affects the physiological and biochemical responses of marine organisms, which are driven by the regulation of gene expression (Hornstein et al., 2018; Rahman et al., 2019; Zhang et al., 2020). Stress-activated protein kinases have been proven to play significant roles in the thermal response of marine organisms (Anestis et al., 2008; Tomanek and Zuzow, 2010; Dong et al., 2011; Yao and Somero, 2012). Mitogen-activated protein kinases (MAPKs) are protein Ser/Thr kinases that transmit extracellular signals and cause many intracellular reactions (Cargnello and Roux, 2011). This process is realized by the cascade reaction of conservative MAPK, mitogen-activated protein kinase (MAPKK), and mitogen-activated protein kinase (MAPKKK). The MAPK mainly has three subfamilies, including extracellular signal regulated kinase (ERK), c-Jun NH_2_-terminal kinase (JNK), and p38 (Johnson and Lapadat, 2002). The classification of subfamily is based on the Thr-X-Tyr sequence of the tripeptide motif: ERK is Thr-Glu-Tyr, JNK is Thr-Pro-Tyr, and p38 is Thr-Gly-Tyr. The numbers of gene copy in different subfamilies are different among species. In vertebrates, *ERK* subfamily includes *ERK1* and *ERK2* (Boulton et al., 1990, 1991); *JNK* subfamily includes *JNK1*, *JNK2*, and *JNK3* (Kyriakis et al., 1994); and *p38* subfamily includes *p38*α, *p38*β, *p38*γ, and *p38*δ (Cuadrado and Nebreda, 2010). There are also some atypical subfamilies, such as ERK3/4 and ERK7/8, whose activation process is not a typical three-level cascade reaction (Lau and Xu, 2019). The MAPK signaling pathway is highly conservative in evolution and closely related to a variety of signal transmission and feedback (Yang et al., 2010). Many studies have found that MAPK played important roles in controlling the response of cells to the environment, extensively participating in various physiological processes such as cell growth, cell differentiation, proliferation, and apoptosis (Johnson and Lapadat, 2002; Morrison, 2012).

Extracellular signal regulated kinase signaling pathway is the first discovered MAPK signaling pathway including ERK1/2 and ERK5. ERK pathway is essential in the regulation of cell proliferation and differentiation in all organisms (Jing and Anning, 2005). In starfish (*Asterina miniata*) and sea urchin (*Strongylocentrotus purpuratus*), ERK pathway can regulate the meiotic process of oocytes (Carroll et al., 2000). In *Chaetopleura*, *Tectura*, and *Lymnaea*, ERK pathway has been proven to participate in the cell division of blastomere (Lambert and Nagy, 2003). In the study of *Ciona intestinalis*, ERK pathway can induce the formation of ascidian notochord precursor (Picco et al., 2007), embryonic nerve, and brain (Akanuma and Nishida, 2004). Besides, many studies showed that ERK pathway also participate in the immune response of organism. For example, ERK was found to be a downstream target of immune signal transmission in the hemocytes of gastropod snail (*Lymnaea stagnalis*), which played a crucial role in the Molluscan innate defense response (Lacchini et al., 2006). The JNK signal pathway is another signaling pathway whose activation site is located in the amino-terminal active region (Weston and Davis, 2007). JNK is also called stress-activated protein kinase (SAPK), and its upstream kinases mitogen-activated protein kinase 4 (MEK4) and mitogen-activated protein kinase 7 (MEK7) are key nodes in the JNK signal pathway (Dhanasekaran and Reddy, 2008). The different functions of JNK1/2/3 mainly depend on the specific site of their binding to the substrate (Ye et al., 2009). The currently known substrates of JNK are Ets-like protein 1 (Elk-1), activating transcription factor 2 (ATF-2), and c-Jun. p38 also belongs to SAPK, which can be activated by many stress conditions and mediates various stress responses (Johnson and Lapadat, 2002). Stresses such as high temperature, hypoxia, and hypertonicity can activate JNK and p38 pathways of *M. edulis* (Gaitanaki et al., 2004). In *Drosophila* hemocytes, JNK signaling pathway mediated the immune response to endotoxin through a rapid activation of JNK during a bacterial lipopolysaccharide (LPS) infection (Sluss et al., 1996). In red swamp crayfish (*Procambarus clarkii*), the expression of *p38* gene regulated the distribution and accumulation of cadmium (Cd) in different tissues under Cd-stressed condition (Shui et al., 2020).

All above studies emphasize the important functions of MAPK signaling pathway in environmental response, cell proliferation and differentiation, etc. However, the knowledge regarding MAPK in invertebrate responding to environmental stress is still unclear, and further researches are necessary. In the present study, we carried out the systematic identification of *MAPK* in the genome of *C. farreri* and phylogenetic analysis and examined their expression profiles during larval development stages, in adult tissues, and under heat stress. The results will contribute to better understand the function of MAPK and provide valuable information for elucidating environmental adaptation of bivalves.

## Materials and Methods

### Genome-Wide Identification and Sequence Analysis of MAPK Genes in *C. farreri*

The whole-genome of *C. farreri* (PRJAN185456) (Li et al., 2017) were used to search with the typical MAPK protein sequences of other species, including ERK (*Caenorhabditis elegans*, *Patinopecten yessoensis*, *Crassostrea gigas*, *Crassostrea virginica*, and *Ciona intestinalis*), JNK (*Apostichopus japonicas*, *Danio rerio*, *Xenopus tropicalis*, and *Gallus gallus*), and p38 (*Mus musculus* and *Homo sapiens*), retrieved from the National Center for Biotechnology Information (NCBI)^1^, Wormbase^2^, and Flybase^3^ (Supplementary Table 1). The open reading frame (ORF) finder was used to predict their gene structure^4^. The amino acid sequences were verified by BLASTP against the NCBI non-redundant protein sequence database. The conserved domains were predicted by SMART^5^. The putative isoelectric point (PI) and molecular weight were predicted by ExPASy-Prot^6^. The secondary structures was predicted by software Geneious 4.8.4^7^.

### Phylogenetic Analysis

Multiple protein sequences alignments were performed on identified MAPK protein sequences from *C. farreri* and other selected organisms using the ClustalW2^8^. The phylogenetic tree was constructed utilizing software MEGA-X with the neighbor-joining (NJ) method (Kumar et al., 2016). Bootstrapping with 5,000 replications was used to evaluate the phylogenetic tree.

### Spatiotemporal Expression of MAPK Genes in *C. farreri*

The expression profiles of *CfMAPKs* were analyzed using RNA-seq datasets (SRX2444844-SRX2444876 and SRX2510167-SRX2510176) of *C. farreri* (Li et al., 2017). The expression level was described by transcripts per kilobase per million mapped reads (TPM). The TPM values from the RNA-seq datasets, including different developmental stages (zygote, multicell, blastula, gastrula, trochophore, D-shaped larvae, early umbo, middle umbo, post umbo, eyespots larvae, and juvenile) and adult tissues (adductor muscle, smooth muscle, foot, mantle, eye, gill, hemolymph, digestive gland, kidney, female gonad, male gonad, cerebral ganglia, and visceral ganglia) were Log_2_ transformed and subsequently used to perform a visualization of expression analysis with heat map by software TBtools (Chen et al., 2020).

### Expression Analysis of *CfMAPKs* Under Heat Stress

The transcriptome databases of *C. farreri* in response to heat stress were independently constructed by our laboratory (unpublished data). In simple terms, 27°C was used as thermal stimulus condition, which is close to the maximum temperature at sea area of scallop distribution, and 20°C was used as control temperature. Transcriptome databases at eight time points (3, 6, 12, and 24 h, 3, 6, 15, and 30 days) in four tissues (three individual replications were set), including mantle, gill, heart and hemolymph, were used to reveal the expression levels of *CfMAPKs* under heat stress. Statistical analyses were conducted with one-way analysis of variance followed by *post hoc* comparison of means bases Tukey honestly significant difference (HSD) with software IBM SPSS Statistics 20; significant level was set at *p* < 0.05.

## Results

### Gene Identification and Sequence Analysis

**FIGURE 1:**
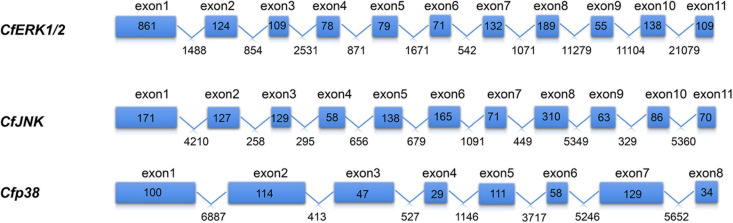
Gene structure of *CfMAPKs*. The blue boxes indicate the exons, and the polylines indicate the introns. The numbers in the boxes indicate the length of exon; the numbers under the line indicate the length of intron.

**FIGURE 2:**
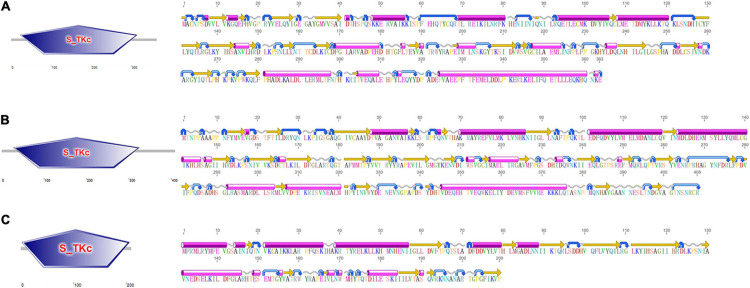
The conserved domain (left) and secondary structure (right) of **(A)** CfERK1/2, **(B)** CfJNK, and **(C)** Cfp38. The pink cylinders stand for alpha helixes, the orange straight arrows stand for beta strands, the gray wavy lines stand for coil, and the blue curved arrows stand for turns.

Three *CfMAPKs* were identified from the transcriptome and genome of *C. farreri*, which showed high sequence similarity with *ERK*, *JNK*, and *p38* of species from invertebrate to vertebrate. Three genes were named separately as *CfERK1/2*, *CfJNK*, and *Cfp38*. As shown in Table 1, the length of *CfERK*, *CfJNK*, and *Cfp38* was 1,962, 2,075, and 630 bp, encoding 363, 408, and 209 amino acids, respectively. *CfERK1/2* and *CfJNK* were composed of 11 exons and 10 introns; *Cfp38* was composed of 8 exons and 7 introns (Figure 1). Some introns of *CfERK* are extremely long, such as the last three introns that were longer than 10 kb (11,279, 11,104, and 21,079 bp). The length of some exons was similar among the three *CfMAPKs*. For example, the length of second exons was similar 124, 127, and 114 bp, respectively. More strikingly, the exon 6 in *CfERK* and exon 7 in *CfJNK* were the same in length (71 bp). Both exon 4 of *CfJNK* and exon 6 of *Cfp38* were of the same length. The conserved domain (S_TKc) in all CfMAPKs was predicted by SMART (Figure 2). The secondary structure showed that all three *CfMAPK* genes have four structures including α helix, β sheet, random coil, and turn. However, the number of different structures among members was pretty different (Table 1 and Figure 2).

**TABLE 1:** Characteristics of the *MAPK* genes in *C. farreri*.

	**ERK1/2**	**JNK**	**p38**
Total length (bp)	1962	2075	630
5′UTR length (bp)	861	291	–
3′UTR length (bp)	9	557	–
ORF length (bp)	1092	1227	630
Amino acids length	363	408	209
Weight (kDa)	42.28	46.25	24.09
Theoretical pI	6.28	6.02	9.49
Number of exons	11	11	8
Number of introns	10	10	7
Number of alpha helixes	21	15	13
Number of beta strands	21	27	12
Number of coils	26	34	14
Number of turns	35	36	15

**FIGURE 3:**
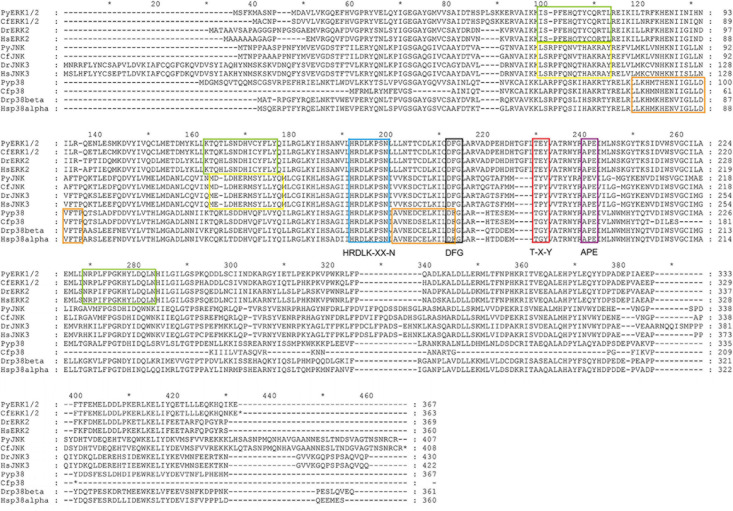
Alignment of the amino acid sequences of CfMAPK with other MAPKs. The blue box indicates the HRDLK-XX-N motif; the black and the purple boxes indicate the DFG and APE motif, respectively; the red box indicates the phosphorylation T-X-Y motif; and the green, yellow, and orange boxes indicate the conserved signature sequences of ERK, JNK, and p38, respectively.

Based on multiple sequences alignment of the MAPK amino acid sequences among *C. farreri* and other species including *H. sapiens*, *D. rerio*, and *P. yessoensis*, the dual phosphorylation T-X-Y motif located and some functional sites, such as HRDLK-XX-N, DFG, and APE motif, were identified, which were similar to the MAPK proteins reported in other species (Figure 3). In addition to the conserved structures shared by most MAPKs, three specific signature sequences were identified in CfERK (marked with green box), and two specific signature sequences were identified in CfJNK (marked with yellow box) and Cfp38 (marked with orange box), respectively.

### Phylogenetic Analysis

**FIGURE 4:**
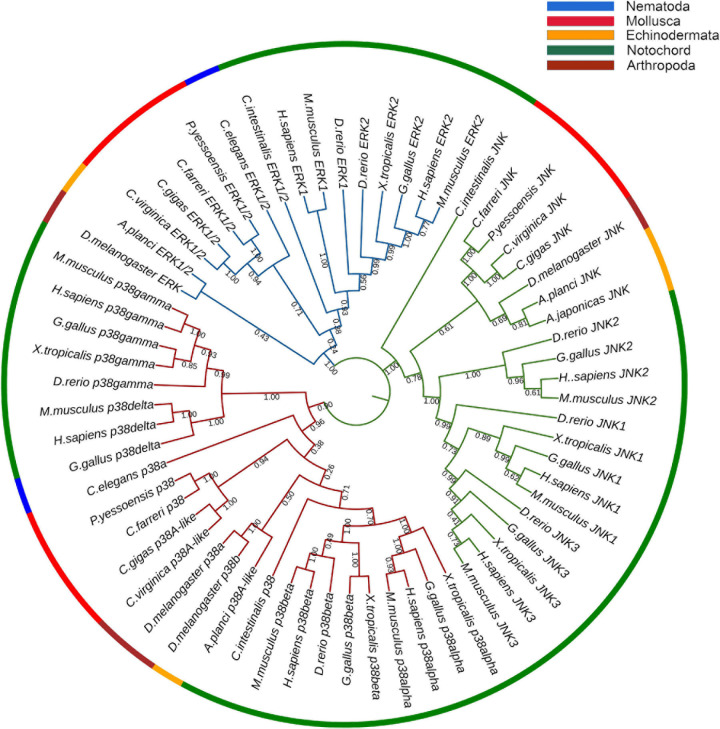
Phylogenetic tree of CfERK, CfJNK, and Cfp38 with other MAPKs. The blue branches are ERK, the green branches are JNK, and the red branches are p38. The colors of the outermost rings represent different phyla, blue represents Nematoda, red represents Mollusca, orange represents Echinodermata, green represents Notochord, and crimson represents Arthropoda. The numbers under the tree branches indicate the bootstrap values from 5,000 replicates.

A NJ phylogenetic tree was constructed using the amino acid sequence of CfMAPKs and MAPK members of other species (Figure 4). As a whole, the phylogenetic tree was classified into three subfamilies, comprising p38, JNK, and ERK, which indicated that ERK, JNK, and p38 were more conserved between homologs of other species than other subfamilies. The three CfMAPKs, respectively, located within one clade and first clustered with MAPKs of other bivalves including *P. yessoensis*, *C. gigas*, and *C. virginica*.

### Spatiotemporal Expression of *CfMAPKs*

**FIGURE 5:**
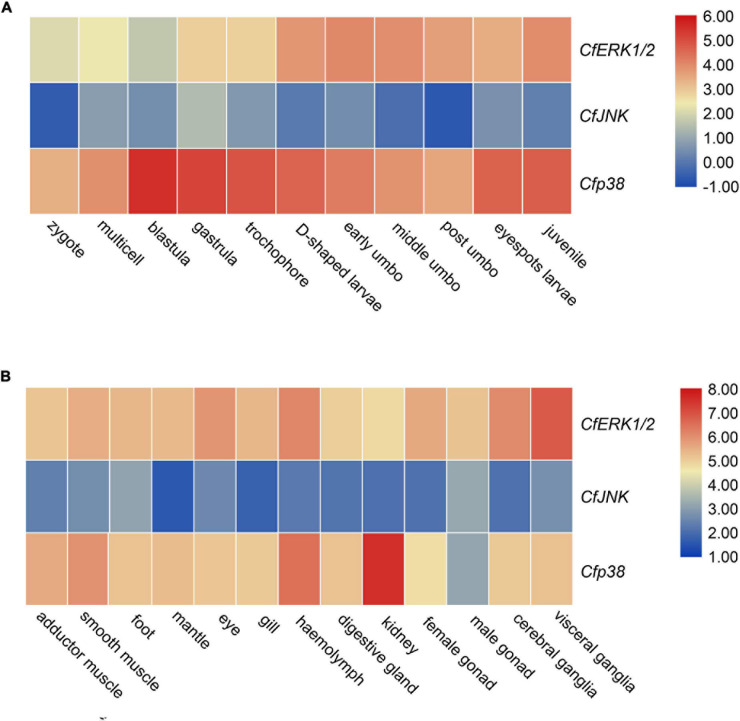
Expression analysis of *CfERK1/2*, *CfJNK*, and *Cfp38* during **(A)** different developmental stages and **(B)** in adult tissues of *C. farreri* based on the log_2_TPM.

RNA-seq datasets for different developmental periods and adult tissues of *C. farreri* were analyzed to detect the expression patterns of *MAPK* genes. During developmental process, *CfERK1/2* and *Cfp38* showed high expression level, but *CfJNK* showed a low expression level (Figure 5A). Specifically, expression of *CfERK1/2* gradually increased from gastrula and maintained a high expression level from D-shaped larvae to juvenile. The *Cfp38* was the highest expression member among *CfMAPKs* and remained continuously express high level during developmental stages, especially blastula, gastrula, trochophore, eyespots larva, and juvenile. In all tested tissues, the expressions of *CfERK* and *Cfp38* were ubiquitous, whereas *CfJNK* was barely expressed (Figure 5B), which showed similar expression pattern with developmental stages. Specifically, *CfERK* showed the highest expression in the visceral ganglia, followed by cerebral ganglia, hemolymph, and eye. *Cfp38* expressed highest in the kidney, followed by hemolymph and smooth muscle.

### Expression Profiles of *CfMAPKs* in Response to Heat Stress

**FIGURE 6:**
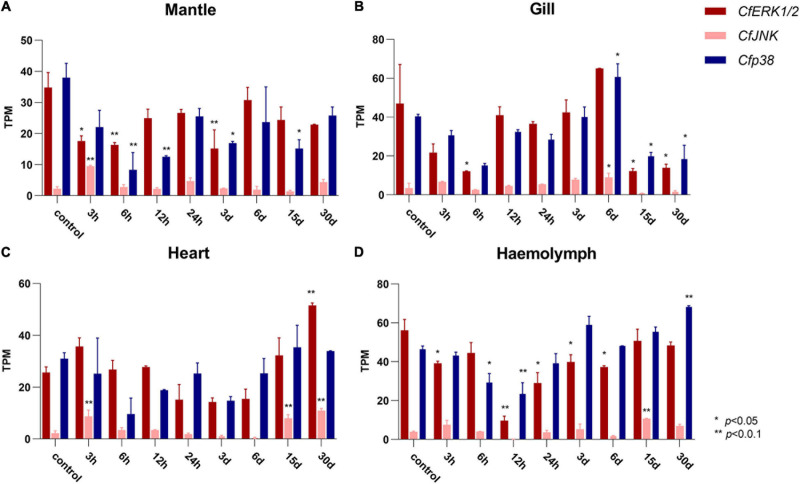
Expression of *CfERK1/2*, *CfJNK*, and *Cfp38* in **(A)** mantle, **(B)** gills, **(C)** heart, and **(D)** hemolymph of *C. farreri* under heat stress at different time points based on the TPM value. The vertical bars represent the mean ± SD (*N* = 3). The expression of *CfMAPKs* at 0 h was used as the control. Values marked with asterisks indicate significant differences from the control expression (*p < 0.05; **p < 0.01).

To examine the expression patterns of *CfMAPK* in response to environmental stresses, RNA-seq datasets from *C. farreri* under heat stress were analyzed. In general, the expression of *CfMAPK* changed apparently and responded frequently in mantle, gill, heart, and hemolymph. Specifically, *CfERK1/2* and *Cfp38* showed general downregulated trends, whereas *CfJNK* upregulated at some time points in the mantle, gill, heart, and hemolymph under heat stress (Figure 6). Comparing to control, *CfERK1/2* declined significantly at 3, 6 h, and 3 days; *Cfp38* declined significantly at 6, 12 h, and 3, 15 days, while *CfJNK* increased markedly at 3 h in mantle (*p* < 0.05). Except for *CfJNK* (only increased significantly at 15 days), the expression pattern of *CfERK1/2* and *Cfp38* in the hemolymph was similar with that in the mantle, just with different time points of significant change. Besides, a down–up–down expression pattern *CfERK1/2* and *Cfp38* was found in gill, while *CfJNK* increased significantly at 6 days. Comparing to other three tissues, the expression of *CfERK1/2* and *Cfp38* was not significantly changed in the heart, while *CfJNK* increased significantly at 3 h, 15, and 30 days. Overall, the expression of *CfERK1/2* and *Cfp38* showed a downregulated tendency, while *CfJNK* was upregulated at some time points under heat stress. Moreover, *CfERK1/2* and *Cfp38* had more significant changes than *CfJNK*, which indicated a different function of *CfMAPKs* in response to heat stress.

## Discussion

Mitogen-activated protein kinase signaling pathway is one of the ancient and evolutionarily conservative signaling pathways from yeast to human, which is involved in a vast array of physiological processes such as growth and development, stress response, and apoptosis (Johnson and Lapadat, 2002; Jing and Anning, 2005; Cargnello and Roux, 2011; Morrison, 2012). Until now, the features and functions of MAPK have been widely reported in vertebrate species, especially in mammals, while limited studies have been carried out in Mollusca. In the present study, based on the whole genome sequences and transcriptome databases of *C. farreri*, we systematically conducted the identification and evolution analysis of *CfMAPK* and examined their expression profiles during larval development stages, in adult tissues, and under heat stress.

In vertebrates, such as *M. musculus* and *H. sapiens*, there are four MAPK signal cascades including ERK1/2, JNK, p38, and ERK5 signaling pathway (Cargnello and Roux, 2011). At the same time, the genes of these pathways generally have multiple copies, such as two homologous *ERK* isoforms (*ERK1* and *ERK2*), three *JNK* isoforms (*JNK1*, *JNK1* and *JNK3*), and four *p38* isoforms (*p38*α, *p38*β, *p38*γ, and *p38*δ) (Boulton et al., 1990, 1991; Kyriakis et al., 1994; Cuadrado and Nebreda, 2010). In the present study, three *MAPK* genes including *CfERK1/2*, *CfJNK*, and *Cfp38* were successfully identified in the genome of *C. farreri*, but *ERK5* gene is absent. In addition, only one copy of *ERK*, *JNK*, and *p38* was detected in *C. farreri* genome. Moreover, all *CfMAPK* genes were conserved with other species in the same subfamily. The similar genes number and copy pattern of *MAPK*s were also reported in other invertebrates, such as *C. elegans* (Galbadage et al., 2016), *P. yessoensis* (Sun et al., 2016), and *C. gigas* (Qu et al., 2016, 2017). Thus, it is likely that only one copy for each of three MAPK subfamilies exists in the common ancestor of vertebrates and invertebrates, and multiple homologous isoforms of *MAPK* genes in vertebrates originated from the result of ancestral gene expansion during the evolution process.

During the developmental stages from zygote to juvenile scallop, *CfERK1/2* and *Cfp38* expressed apparently higher than *CfJNK*, which indicated that *CfERK1/2* and *Cfp38* may be more involved in the regulation of growth and development in *C. farreri*. *CfERK* maintained a relatively high level from D-shaped larvae to juvenile stages, indicating that it may be involved in the organ development and metamorphic process of *C. farreri* larvae, such as the disappearance of the velizer, the development of the gills and foot, and the formation of the secondary shell. In previous researches, the expression of *ERK* was essential for the tail degeneration of *C. intestinalis* (Chambon et al., 2002). During zebrafish embryonic development, *ERK* was activated by fibroblast growth factor (FGF), and it could regulate the development and formation of organs, including eye, somites, and limbs (Wong et al., 2019). In addition, in the study of *Schistosoma mansoni*, Ressurreição et al. (2011) reported that *p38* of embryo significantly increased 3.7-fold after 28 h larval culture, and activated *p38* was associated with regions of the tegument, neural mass, and germinal cells. In our study, similar expression pattern of *p38* was observed in the development of *C. farreri*, and the expression of *Cfp38* was mainly higher in blastula, gastrula, and trochophore than other stages, suggesting that *p38* may be involved in blastocyst formation and layer differentiation of bivalve. These expression patterns revealed diverse regulation of *CfMAPKs* during developmental stages, which may suggest their fundamental role in larval growth and development of bivalve.

Many studies have found that MAPK played important roles in various physiological processes (Johnson and Lapadat, 2002; Jing and Anning, 2005; Cargnello and Roux, 2011; Morrison, 2012). In our study, *CfMAPKs* were ubiquitously expressed in all the investigated tissues, and the expression levels of *CfERK* and *Cfp38* were higher than that of *CfJNK*, which indicated that *CfERK* and *Cfp38* could play an important role in the biological processes of *C. farreri*. In previous studies, Napoli et al. (2012) reported that ERK signaling could orchestrate nerve repair in Schwann cells of mice after suffering from damage of the peripheral nerves. Ciccarelli and Giustetto (2014) found that ERK governed the transmission of neuronal intracellular signaling between pre- and postsynaptic targets related to regulators of gene expression such as transcription factors and histone proteins to control transcription. These studies demonstrated that *ERK* may be involved in the nerve function and signal transmission. In our study, *CfERK* was specifically highly expressed in the ganglia, which suggested its potentially regulated role in the nerve function of bivalves. Previous researches have shown that MAPK signaling play an important role in regulating the innate immunity. In Nile tilapia (*Oreochromis niloticus*), ERK signaling regulates proliferation of primordial T cells to participate in adaptive immune response during bacterial infection (Wei et al., 2020). In the clam (*Meretrix petechialis*), Zhang et al. (2019) reported that *p38* participated in clam immunity by activating microphthalmia-associated transcription factor (MITF) to regulate the expression of the immune-related gene phenoloxidase (PO). In *P. yessoensis*, the expression of *ERK* and *JNK* significantly upregulated after infection with bacteria, revealing the involvement of *ERK* and *JNK* in defense against bacterial infection (Sun et al., 2016). In our study, *CfERK* and *Cfp38* was highly expressed in hemolymph, known to have an important role in the immune system. In addition, Tian et al. (2020) reported that MAPKs mediated the immune defense ability of *C. farreri* hemocytes after Benzo[a]pyrene exposure. Hence, high expression of *ERK* and *p38* may be related to the immune function of the bivalve. In addition, *Cfp38* was significantly expressed in the kidney. For filter-feeding bivalve, the kidney has not only the function of secretion and excretion but also the function of storage, such as the accumulation of heavy metal ion in *M. galloprovincialis* and the accumulation of potent neurotoxins in *C. farreri* (Wilbur and Yonge, 2013; Li et al., 2017; Wakashin et al., 2018). Thus, high expression of *Cfp38* in the kidney may better protect bivalve from accumulation of toxins by modulating the defense mechanisms, which revealed a molecular adaptation to lifestyle.

To provide insight into the function of *CfMAPKs* in response to environmental stress, the expression of *CfMAPKs* was, respectively, examined in four adult tissues after exposure to heat stress. Temperature is an important environmental factor affecting the physiological and biochemical responses of organisms, and stress caused by sudden changes in temperature interrupts cellular homeostasis, metabolism, and growth (Liu et al., 2004; Hornstein et al., 2018; Rahman et al., 2019; Zhang et al., 2020). Previous studies have shown that the level of MAPK pathway could be induced by cold and heat stress in the gill tissue, mantle tissue, and hemocytes of mussels (Anestis et al., 2008; Yao and Somero, 2012; Wang et al., 2018). In *Takifugu fasciatus*, the MAPK pathways on muscles were also activated under cold stress, including ERK, JNK, and p38, and it could regulate lipid metabolism in response to cold stress (Chu et al., 2021). In the study of chicken fibroblast cells, MAPK pathway genes (ERK, JNK, and p38) were downregulated under heat stress condition, and then, curcumin has the ability to ease the oxidative damage through activating the MAPK signaling pathway (Wu et al., 2019). In general, the expression of *CfERK1/2* and *Cfp38* significantly downregulated and then upregulated in four tissues under heat stress. These results indicated that the heat stress significantly affected the MAPK signaling pathway, which was involved in various tissues of *C. farreri*. In terms of time of thermal stimulation, the expression of *CfMAPK* was inhibited in the short term but gradually recovered in the long term. This unique expression pattern of *MAPKs* suggested a molecular mechanism for ectotherms such as bivalve to adapt to temperature changes. In addition, we also found a special phenomenon that the expression of *CfJNK* in all tissues could be increased within 3 h, which may be an acute stress mechanism of bivalve, and this was the first report in aquatic animals.

In summary, three *MAPK*s, including *CfERK*, *CfJNK*, and *Cfp38*, were successfully identified from the genome of *C. farreri*, and only one copy of *ERK*, *JNK*, and *p38* was detected, respectively. During larval developmental stages and in adult tissues, *CfERK1/2* and *Cfp38* expressed apparently higher than *CfJNK*, which indicated that *CfERK1/2* and *Cfp38* may be more involved in the regulation of growth, development, and physiological processes in *C. farreri*. Under heat stress, the expression of *CfERK1/2* and *Cfp38* were significantly downregulated and then upregulated in four tissues, while the expression of *CfJNK* increased in all tissues; these different expression pattern suggested a different molecular mechanism of *CfMAPKs* for bivalve to adapt to temperature changes. The findings of this study provide insight into understanding the diverse functions of *CfMAPKs* and elucidating MAPK evolution in invertebrates. Further investigation on the functions of *CfMAPKs* will provide a more detailed explanation about the molecular adaptation mechanisms of bivalve under environmental stress.

## Data Availability Statement

The original contributions presented in the study are included in the article/Supplementary Material, further inquiries can be directed to the corresponding author/s.

## Author Contributions

ZL and ZY: conceptualization, methodology, data curation, investigation, formal analysis, visualization, writing – original draft, and writing – review and editing. XH: conceptualization, writing – original draft, writing – review and editing, supervision, project administration, and funding acquisition. CP: methodology, investigation, data curation, formal analysis, and visualization. HY, CC, YH, and XW: methodology, data curation, investigation, fanalysis, and visualization. QX and JH: conceptualization, supervision, and project administration. ZB: supervision, project administration, and funding acquisition. All authors contributed to the article and approved the submitted version.

## Conflict of Interest

The authors declare that the research was conducted in the absence of any commercial or financial relationships that could be construed as a potential conflict of interest.

## Publisher’s Note

All claims expressed in this article are solely those of the authors and do not necessarily represent those of their affiliated organizations, or those of the publisher, the editors and the reviewers. Any product that may be evaluated in this article, or claim that may be made by its manufacturer, is not guaranteed or endorsed by the publisher.

## Footnotes

1
https://www.ncbi.nlm.nih.gov/guide/proteins/

2
https://wormbase.org/

3
http://flybase.org/

4
http://www.ncbi.nlm.nih.gov/orffinder/

5
http://smart.embl.de/

6
https://web.expasy.org/protparam/

7
http://www.geneious.com/

8
http://www.ebi.ac.uk/Tools/msa/clustalo/
