# Supplementary material for: Identification, Characterization, and Expression Analysis Reveal Diverse Regulated Roles of Three MAPK Genes in Chlamys farreri Under Heat Stress
Source: Front Physiol. 2021 Jul 28;12:688626. doi: 10.3389/fphys.2021.688626 (PMC8356821; doi:10.3389/fphys.2021.688626)
Supplement: Supplementary file 1 [file Table_1.docx]

Table S1 Accession number of MAPKs in other species used in this study

| Species | Gene | Accession number |
| --- | --- | --- |
| *Caenorhabditis elegans* | ERK1/2  p38a | WBGene00003401 (Wormbase)  WBGene00004055 (Wormbase) |
| *Patinopecten/Mizuhopecten yessoensis* | ERK1/2  JNK  p38 | ANG60946.1  ANG60947.1  ANG60948.1 |
| *Crassostrea gigas* | ERK1/2  JNK  p38A-like | XP_011437814.1  XP_011442365.1  NP_001295784.1 |
| *Crassostrea virginica* | ERK1/2  JNK  p38A-like | XP_022341056.1  XP_022318851.1  XP_022297910.1 |
| *Drosophila melanogaster* | JNK  p38a  p38b | AAC47325.1  FBpp0083965(Flybase)  FBpp0080111(Flybase) |
| *Apostichopus japonicas* | JNK | PIK60420.1 |
| *Acanthaster planci* | ERK1/2  JNK  p38A-like | XP_022101090.1  XP_022090324.1  XP_022095984.1 |
| *Ciona intestinalis* | ERK1/2  JNK  p38 | NP_001071697.1  NP_001071750.1  NP_001071958.1 |
| *Danio rerio* | ERK1  ERK2  JNK1  JNK2  JNK3  p38-β  p38-γ | NP_958915  NP_878308  NP_001103859  XP_001919688  NP_001032790  NP_001002095  NP_001038306 |
| *Xenopus tropicalis* | ERK2  JNK1  JNK3  p38-β  p38-γ  p38-α | NP_001017127.1  NP_001123415.1  AAI52032.1  NP_001098754.1  NP_001017080.1  NP_001005824.1 |
| *Gallus gallus* | ERK2  JNK1  JNK2  JNK3  p38-β  p38-γ  p38-δ  p38-α | NP_989481  XP_421650.2  NP_990426  XP_420551  NP_001006227  XP_001233062  XP_001234443  XP_001232616 |
| *Mus musculus* | ERK1  ERK2  JNK1  JNK2  JNK3  p38-β  p38-γ  p38-δ  p38-α | NP_036082  NP_036079  NP_057909  NP_001157143  NP_001075036  NP_035291  NP_038899  NP_036080  NP_036081 |
| *Homo sapiens* | ERK1  ERK2  JNK1  JNK2  JNK3  p38-β  p38-γ  p38-δ  p38-α | AAH13992  NP_002736  NP_002741.1  AAH32539.1  NP_002744  NP_002742  NP_002960  NP_002745  NP_001306 |
