# Supplementary material for: Identification, Characterization, and Expression Analysis Reveal Diverse Regulated Roles of Three MAPK Genes in Chlamys farreri Under Heat Stress
Source: Front Physiol. 2021 Jul 28;12:688626. doi: 10.3389/fphys.2021.688626 (PMC8356821; doi:10.3389/fphys.2021.688626)
Supplement: Supplementary file 4 [file Table_4.doc]

>C.farreri ERK1/2

MACNPSDVVLVKGQEFHVGPRYVELQYIGEGAYGMVVSAIDTHSPQSKKERVAIKKISPF

EHQTYCQRTLREIKILNRFKHENIINIQNILRQETLECMKDVYIVQCLMETDMYKLLKTQ

KLSNDHICYFLYQILRGLKYIHSANVLHRDLKPSNLLLNTTCDLKICDFGLARVADPEHD

HTGFLTEYVATRWYRAPEIMLNSKGYTKSIDVWSVGCILAEMLINRPLFPGKHYLDQLNH

ILGILGSPHADDLCSIVNDKARGYIQTLPHKPKVPWKQLFPHADLKALDLLERMLTFNPH

KRITVEQALEHPYLEQYYDPADEPVAEEPFTFEMELDDLPKERLKELIFQETLLLEQKHQ

NKE*

>C.farreri JNK

MTNPPAAAPPNFYMVEVGDSTFTILDRYQNLKPIGSGAQGIVCAAYDTVAGANVAIKKLS

RPFQNVTHAKRAYREFVLMKLVNHKNIIGLLNAFTPQKTLEDFQDVYLVMELMDANLCQV

INMDLDHERMSYLLYQMLCGIKHLHSAGIIHRDLKPSNIVVKSDCTLKILDFGLARTQGT

AFMMTPYVVTRYYRAPEVILGMGYKENVDIWSVGCIMAELIRGAVMFPGSDHIDQWNKII

EQLGTPSREFMQRLQPTVRSYVENRPRHAGYNFDRLFPDVIFPQDSADHSGLRASMARDL

LSRMLVVDPEKRISVNEALMHPYINVWYDENEVNGPAPDSYDHTVDEQEHTVEQWKELIY

DEVMSFVVREKKKLQTASNPMQNHAVGAANNESLTNDGVAGTNSNRCR*

>C.farreri p38

MFRMLRYMFEVGSAINIQTNVKCAIKKLARPFQSKIHAKRTYRELKLLKHMNHENIIGLL

DVFTPQTSLADFDDVYLVTHLMGADLNNIIKTQRLSDDHVQFLVYQILRGLKYIHSAGII

HRDLKPSNIAVNEDSELKILDFGLARHTESEMTGYVATRWYRAPEIVLNWMHYTQTDILE

SKIIILVTASQVRKNNANARTGPGFIKVP*
